# QTL associated with Gummy Stem Blight (GSB) resistance in watermelon

**DOI:** 10.1186/s12864-022-08849-2

**Published:** 2022-09-03

**Authors:** Jeong-Eui Hong, Mohammad Rashed Hossain, Hee-Jeong Jung, Ill-Sup Nou

**Affiliations:** 1 Department of Horticulture, Sunchon National University, 255 Jungang-ro, Suncheon, Jeonnam 57922 Korea; 2 Department of Genetics and Plant Breeding, Bangladesh Agricultural University, Mymensingh, 2202 Bangladesh

**Keywords:** Watermelon, QTL, Gummy stem blight

## Abstract

**Background:**

Gummy stem blight (GSB), caused by *Didymella bryoniae* (syn. *Stagonosporopsis cucurbitacearum*), produces devastating symptoms on whole plants of watermelon (*Citrullus lanatus*) and other cucurbits, significantly reducing yield and quality. Identification of genetic determinants and sources of resistance to this devastating GSB disease in watermelon is essential for developing resistant varieties.

**Results:**

In this study, we aimed at identifying quantitative trait loci (QTLs) linked to GSB resistance in melon. We identified the genome-wide single nucleotide polymorphisms (SNPs) by genotyping by sequencing (GBS) of an F_2_ population developed from *C. lanatus* lines, ‘PI 279461’ (*resistant*) ✕ ‘PI 223764’ (susceptible). Inheritance analysis indicated that resistance to GSB is a multi-genic trait in this population. Three QTLs namely, *ClGSB1.1*, *ClGSB10.1*, and *ClGSB11.1* associated with GSB resistance, explaining approximately 10% of the phenotypic variation, were identified. Among these, the QTL *ClGSB1.1* on chromosome 1 is identified as a major QTL harboring five candidate genes associated with GSB resistance including two RLKs (*ClC01G014900* and *ClC01G015010*), two WRKY transcription factors (*ClC01G014910* and *ClC01G014990*), and one AvrRpt-cleavage domain protein (*ClC01G015130*).

**Conclusion:**

Two high resolution melting (HRM) markers, *WmGSB1.1–2* and *WmGSB1.1–7* having a high positive correlation with the phenotypic variations, were developed. Five potential candidate genes were predicted to be associated with GSB resistance. These findings will help breeders to develop watermelon cultivars resistant to GSB.

**Supplementary Information:**

The online version contains supplementary material available at 10.1186/s12864-022-08849-2.

## Introduction

Watermelon (*Citrullus lanatus*) is one of the most popular fruits in the Cucurbitaceae family, and is grown throughout tropical to temperate regions of the world where the climate is favorable (FAOstat 2021). Watermelon fruit is rich in water (91%), and important nutritional compounds such as sugars, lycopene, β-carotene and citrulline, which are very beneficial to human health [1].

Watermelon production is frequently hampered by various insects and diseases, of which gummy stem blight (GSB), caused by the soil, airborne, and seed-borne fungal pathogen *Didymella bryoniae* (syn. *Stagonosporopsis cucurbitacearum*), is the most devastating disease of watermelon [2–4]. GSB symptoms include circular dark tan lesions that blight the leaf, stem cankers, and gummy brown ooze exuding from cankers. Although chemical control methods had been moderately effective in controlling GSB, the repeated use of chemicals has a negative impact on the environment, and may become ineffective due to the rise of resistance to chemicals in certain pathogenic isolates [5, 6]. Therefore, development of resistant cultivars is the most eco-friendly, cost-effective and sustainable method of watermelon production.

Several sources of genetic resistance to GSB have been identified, mostly in the wild relative, *Citrullus amarus* [7, 8] and later, in both *C. amarus* and *C. lanatus* [9, 10]. Genetic studies of these resistant genotypes indicated various patterns of genetic control of resistance to GSB, including monogenic control in PI 189225 [11] and polygenic control with minor effects from individual genes in PI 189225, PI 482283, and PI 526233 [4, 12, 13]. Several studies have mapped the QTLs underlying GSB resistance in watermelon, including one QTL on chromosome 8 of PI 189225 explaining 32% of the phenotypic variations [13]; three QTLs on chromosomes 3, 5 and 7, explaining between 6.4 and 21.1% of the phenotypic variations [14] and another three QTLs on chromosomes 8 and 6 [15]. Besides, QTLs for GSB resistance have also been identified in other cucurbits, such as Cucumber [16, 17] and melon [12, 18].

The release of draft genomes of watermelon and genotyping by sequencing (GBS) has made it possible to discover genome-wide sequence variations including single nucleotide polymorphism (SNP) and Insertion/Deletion (InDel) especially in the causal genes within the QTL regions [19, 20]. This facilitated the development of high throughput molecular markers such as Cleaved amplified polymorphic sequence (CAPS) markers, high-resolution melting (HRM) and PCR amplicon using InDel based markers in *Cucurbitaceae*. CAPS/HRM markers have been reported for ‘PMR 5’ resistance to powdery mildew race 5 in melon [21]. GBS and ddRAD-seq techniques have been used for mapping genes and development of markers in several plant species such as rapeseed, soybean, maize, strawberry and melon [22–25]. Among these assays, HRM and Kompetitive allele specific PCR (KASP) assays have relatively simple, fast, and cost-effective approaches. Recently, high throughput KASP markers for resistance to GSB have been reported in watermelon [13–15]. However, these markers were developed based on the resistance of *C. amarus* (PI 482276 and PI 189225) and hence, are often not effective on *C. lanatus* cultivars. Therefore, identification of QTLs conferring resistance to prevalent pathogenic *D. bryoniae* strains of different parts of the world and development of effective high-throughput markers based on *C. lanatus* are essential for any marker-assisted future breeding endeavors. This study describes the identification of QTL and development of HRM markers using *C. lanatus* accessions against the devastating Korean isolate of *D. bryoniae* in watermelon.

## Materials and methods

### Plant materials and population development

The F_2_ segregating population of 128 plants were generated from the cross of the resistant watermelon (*C. lanatus*) genotype, PI 279461 (♀) and the susceptible genotype, PI 223764 (♂). In addition, another seven PI accessions were obtained from the U.S. National Plant Germplasm System (https://npgsweb.ars-grin.gov/gringlobal/search), U.S. Department of Agriculture (USDA), USA, and one accession, SNWK108 was obtained from Sunchon National University, Korea. All watermelon plants were grown in plant culture room at 24–28 °C and 60% relative humidity under long-day conditions (14-h light/10-h dark cycles).

### Pathogen culture and inoculation

The fungal isolate *Didymella bryoniae,* 13–020, was obtained from the National Institute of Horticultural and Herbal Science (NIHHS) in Korea [12]. The fungus was grown on potato dextrose agar (PDA) medium for 2 weeks at 24 ± 2 °C until the formation of pycnidia [12]. The inoculum was prepared at a final concentration of 5 × 10^5^ spores/mL in deionized water using a hemocytometer. Inoculation was done using a hand-spray bottle when the second true leaves of the plants were fully open. The inoculated plants were then incubated in a plastic tent to maintain high relative humidity [12, 26]. Re-inoculation was performed after 3 days of first inoculation to achieve successful inoculation of all plants and to eliminate false positives.

### Disease assessment

**Fig. 1:**
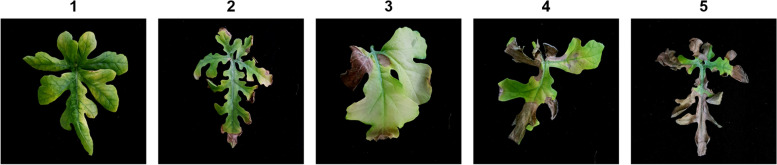
Disease scores used to estimate the severity of gummy stem blight caused by *Didymella bryoniae* at 2 weeks after inoculation (WAI). Leaves were rated according to the following scales: 1 = 0–1% affected leaf area, 2 = 1–10%, 3 = 11–30%, 4 = 31–50%, and 5 = 51–100% and death of plant

Each inoculated plant was scored at 2 weeks after inoculation (WAI) using a scale of 1 to 5: 1, < 1% affected leaf area; 2, 1–10% affected leaf area; 3, 11–30% affected leaf area; 4, 31–50% affected leaf area; and 5, 51–100% affected leaf area and death of plant (Fig. 1). The percentage of inoculated area was measured by the ratio of inoculated to total leaf area, multiplied by 100. The percent disease index (PDI) was calculated to reliably identify phenotypic data using the following equation:

$$\mathrm{PDI}=100\times \sum \frac{\mathrm{Sum}\ \mathrm{of}\ \mathrm{numerical}\ \mathrm{disease}\ \mathrm{rating}}{\ \mathrm{Number}\ \mathrm{of}\ \mathrm{plants}\ \mathrm{evaluated}\times \mathrm{maximum}\ \mathrm{disease}\ \mathrm{rating}\ \mathrm{score}\ }$$

Individulas with a PDI of ≤ 30 (when leaves of the inoculated plant have few symptoms) and > 30 were considered as resistant and susceptible, respectively.

### Extraction of genomic DNA and HRM assay

Genomic DNA (gDNA) was extracted from the young leaves using the DNeasy Plant Mini Kit (QIAGEN, Hilden, Germany) as per the manufacturer’s instructions. The concentration of DNA was quantified by a ND-100 Micro- spectrophotometer (NanoDrop Technologies Inc., Wilmington, DE, USA). To detect SNPs, HRM curve analysis was combined with a 3-blocked and unlabeled oligonucleotide probe (HybProbe) specific to the SNP site [21]. SYTO 9 green fluorescent nucleic acid stain (Invitrogen, Thermo Fisher Scientific, USA) was used in PCR to generate melting curves specific to the probe genotype. Primer and probe were synthesized by Macrogen Co., Seoul, Korea. The HRM PCR mixture contains 1 μl of genomic DNA at 5 ng/μl, 0.1 μl forward and 0.5 μl reverse primers (10 pmol), 0.5 μl probe (10 pmol), 0.3 μl SYTO 9 fluorescent dye, 5 μl HS prime LP premix (GENETBIO, Daejeon, Korea), and 2.6 μl DDW in a total volume of 10 μl. Primers are listed in Table S1. The PCR was performed as: a 5 minute initial pre-incubation at 95 °C followed by 45 cycles of 95 °C for 20 seconds, annealing at 64 °C and 56 °C for 20 s under touchdown command, and 72 °C for 20 s. At the final step, four readings per °C were taken after 60 seconds at 95 °C, 120 seconds at 40 °C, and 1 s at 97 °C. HRM curve analysis was performed using LightCycler 96 software version 1.1 (Roche, Mannheim, Germany) at 100% discrimination for both delta Tm and curve shape with a 0.02 positive/negative threshold level.

### Genotyping by sequencing and QTL mapping

For genotyping by sequencing (GBS), samples of a total of 96 plants (two plants of each of the parents; two plants of the F_1_ generation; 45 resistant and 45 susceptible plants from the F_2_ generation) were prepared [27, 28]. QIAquick PCR Purification Kit (QIAGEN, Hilden, Germany) was used to purify the samples according to the manufacturer’s instructions. Genomic DNA sequencing was carried out at the Macrogen Co., Seoul, Korea, through the HiseqX instrument (Illumina, San Diego, USA). GBS was performed for the genomic DNA sequence of PI 223764, PI 279461, F_1_ generation and F_2_ generation with a custom designed method [29] by DNACARE, Seoul, Korea (Table S2). Using Burrow-Wheeler Aligner (BWA), pair end reads of each parent were aligned to the reference genome (97,103 watermelon genome version 2) [30, 31]. GATK Hablotypecaller-GVCF genotyper pipeline was used to SNP call variants which was then filtered by VCF tools (MQ < 40 and FS > 60) [32]. The SNPs were filtered and selected with the following characteristics: (i) homozygous SNPs in parent lines and heterozygous SNPs in F_1_ population, (ii) SNPs with A/G, A/C, T/G, and T/C combinations, excluding A/T and G/C, (iii) a criterion of a genotype missing < 40%, (iv) maintaining 100 kb of spacing between markers, and (v) segregation distortion at *P* < 0.001.

A genetic linkage map was constructed using JoinMap software version 4.1 (Kyazma, Wageningen, Netherlands) using the Kosambi mapping function, and they were plotted on the 11 linkage groups based on their physical and genetic distance. QTL mapping was used to identify the position of QTL for GSB resistance traits using inclusive composite interval mapping conducted in QTL ICIMapping software version 4.2 [33]. Significant thresholds with 1000 permutations were found in stepwise regressions with *p* < 0.001. The location of the QTL was described using the LOD (logarithm of odds) value. The contribution rate (PVE) was calculated by dividing the percentage of variance explained by each QTL by the total phenotypic variance. The additive effect was estimated to determine whether there was a positive or negative effect on the target trait. Putative candidate genes within significant QTLs were identified from the 97,103 genome v2 . Syntenic regions associated with GSB resistance in other cucurbits were identified using the Search Synteny Blocks of the CuGenDB (http://cucurbitgenomics.org) [34].

### RNA extraction and qRT-PCR analysis

Total RNAs were extracted from 100 mg of powdered watermelon leaves inoculated for 0 hours (control), 12 hours, 24 hours, and 72 hours using a MiniBEST Plant RNA Extraction Kit (TaKaRa, Seoul, Korea) according to the manufacturer’s instructions. Total RNA concentration and quality were measured using a ND-100 Micro-spectrophotometer. A first-strand synthesis kit (Enzynomics, Daejeon, Korea) with oligo (dT) primers was used for cDNA synthesis from 1 μg of total RNA. The cDNA was then used for real time quantitative PCR with a LightCycler 96 instrument using qPCRBIO SyGreen Mix (PCR Biosystems, London, UK). The qRT-PCR conditions were as follows: 95 °C for 5 min; 3-step amplification at 95 °C for 15 second, 58 °C for 15 s and 72 °C for 20 s for 45 cycles. Primers are listed in Supplementary Table 3. The relative expression level of each gene was calculated by 2^- ΔΔct^ method [35], using *Actin* (*ClC02G026960*) as an internal control [36].

### Statistical data analysis

Using the XLSTAT software, a chi-square (2) test for goodness-of-fit was used to determine deviations of observed data from expected segregation ratios. The data is presented as the mean ± standard error of the mean. The student’s t-test or one-way ANOVA were used to assess statistical differences. A *p*-value of 0.05 was used to determine the significance of differences between means. PRISM 6 software was used for all analyses (ver. 6.01, GraphPad Inc., San Diego, USA).

## Results

### Inheritance of GSB resistance in watermelon

**Fig. 2:**
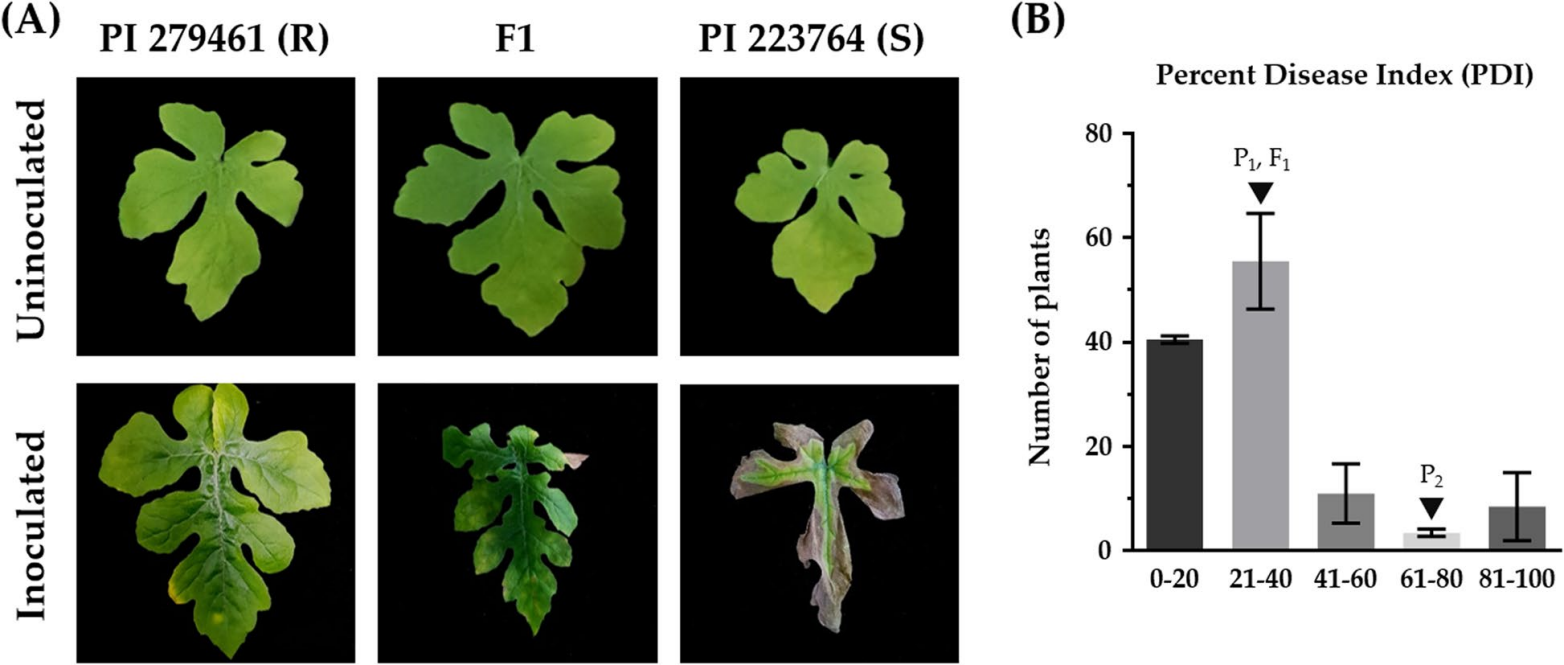
**A** Phenotypes of the resistant (PI 2794461), susceptible (PI 223764), and their F_1_ generation two-weeks after inoculation with *D. bryoniae*. **B** Frequency distribution of disease severity in F_2_ population. P_1_, P_2_ and F_1_ indicate PI 279461, PI 223764 and their hybrids, respectively. Error bars indicate S.E.M.

The average (of 10 individuals) percent disease index (PDI) of the resistant parent, PI 279461 (24%) and the F_1_ plants (26%) were significantly lower compared to that of the susceptible parent, PI 223764 (85%) (Fig. 2A). Among the 128 F_2_ genotypes, 85 were resistant while 43 were susceptible (Table S2 and Fig. S1). Chi-square (χ^2^) test revealed that resistance to GSB doesn’t follow a 3:1 (resistant: susceptible) segregation ratio (Chi-square value of 5.04 at *p* < 0.05). In addition, the frequency distribution of the PDI of F_2_ population doesn’t show a normal distribution either (Fig. 2B). These suggest the presence of a quantitative mode of inheritance of resistance to GSB in the F_2_ population indicating that the resistance trait is most likely to be controlled by multiple genes.

### Genetic map construction and QTL mapping

**Fig. 3:**
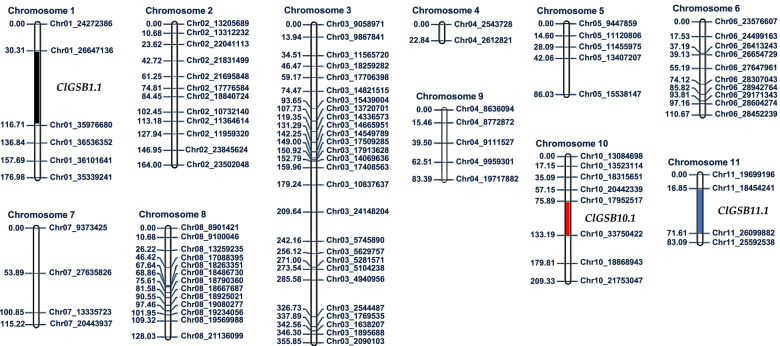
Genetic map of watermelon constructed by using the identified SNPs via GBS analysis. The numbers on the left side represent the g enetic distance from the top of each chromosome in centi Morgan (cM). Names of the right side indicate SNP marker name. QTLs, *ClGSB1.1*, *ClGSB10.1* and *ClGSB11.1* obtained by composite interval mapping (CIM) analysis are shown in black, red and blue boxes on chromosome 1, 10 and 11, respectively

Genotyping by sequencing (GBS) analysis of parental, F_1_ and F_2_ populations identified a total of 593,024 SNPs and InDels polymorphisms between resistant vs susceptible genotypes (Table S4). A genetic map was constructed using 90 F_2_ plants segregating for GSB resistance, and 92 filtered markers (Fig. 3). The total length of the genetic linkage map was 1434.2 cM, with an average length of 130.4 cM and an average of 9.6 markers per linkage group. The average interval between markers was 18.11 cM (Fig. 3 and Table S5). A total of three QTLs for resistance to GSB namely, Cl*GSB1.1* on chromosome 1 (89.5–117.5 cM), *ClGSB10.1* on chromosome 10 (98.5–115.5 cM) and *ClGSB11.1* one chromosome 11 (36.5–52.5 cM) with maximum LOD scores of 6.66, 6.05 and 6.55, respectively, were identified via composite interval mapping (Fig. 3 and Table 1). The genes within each of the three QTLs *(*1209 within *ClGSB1.1*, 1225 within *ClGSB10.1* and 348 within *ClGSB11.1*) were identified as shown in Table S6.

**Table 1:** Details of the QTLs identified for resistance to GSB in the F_2_ populations derived from the cross of PI 279461 (♀) and PI 223764 (♂)

QTL name	Chromosome	Peak (cM)	LOD^**a**^	Add^**b**^	Dom^**c**^	PVE^**d**^	2 – LOD interval (cM)^**e**^	Left flanking Marker	Right flanking Marker
*ClGSB1.1*	1	105	6.66	−1.9181	2.0808	10.237	89.5–117.5	26,647,136	35,976,680
*ClGSB10.1*	10	107	6.05	1.9008	2.099	10.245	98.5–115.5	17,952,517	33,750,422
*ClGSB11.1*	11	45	6.55	−1.9212	2.0783	10.236	36.5–52.5	18,454,241	26,099,882

^a^*LOD* Logarithm of odds ratios at the position of the peak, ^b^*Add* Additive effect of QTL, ^c^*Dom* Dominance effect of QTL, ^d^*PVE* Percent of phenotypic variance explained by the QTL, ^e^*LOD* The QTL interval on genetic map

### Development of HRM markers using identified SNPs in QTLs

To develop high throughput HRM (high-resolution melting) markers, 60, 65, and 11 SNPs/InDels were identified in the QTLs of chromosomes 1, 10, and 11, respectively. Among these, 9, 3, and 4 SNP markers on chromosomes 1, 10, and 11, respectively, were selected based on the high agreement of GBS data based genotype and phenotype in P_1_, P_2_, F_1_ and F_2_ (Table 2 and Table S7). Five SNPs: *WmGSB1.1–3*, *WmGSB1.1–5, WmGSB1.1–6, WmGSB1.1–7* and *WmGSB10.1–2* were found in intragenic regions and the others in intergenic regions (Table 1). These markers were first validated in parental and F_1_ generation (Fig. S2). *WmGSB1.1–2*, *WmGSB1.1–3*, *WmGSB1.1–4*, *WmGSB1.1–5*, *WmGSB1.1–6*, *WmGSB1.1–7*, *WmGSB1.1–9*, *WmGSB10.1–1*, and *WmGSB10.1–2* showed polymorphic melting curves between resistant and susceptible parents, as well as a heterogeneous melting curve in the F_1_ generation (Fig. S2). The marker *WmGSB1*.*1–8* identified polymorphism in the parental lines, but failed to show the heterogeneity in the F_1_ generation.

**Table 2:** Genomic location of SNPs/InDel and their nucleotide variation with the watermelon reference genome

QTL	Chromosome	SNP name	SNP position		Ref	PI 223764 (S)	PI 279461 (R)
***ClGSB1.1***	Chr01	*WmGSB1.1–1*	26,647,136	Intergenic region	A	G/G	A/A
	Chr01	*WmGSB1.1–2*	28,572,939	Intergenic region	C	T/T	C/C
	Chr01	*WmGSB1.1–3*	29,405,595	Intragenic region (Intron)	T	C/C	T/T
	Chr01	*WmGSB1.1–4*	33,392,571	Intergenic region	A	A/A	T/T
	Chr01	*WmGSB1.1–5*	35,339,241	Intragenic region (Exon)	G	A/A	G/G
	Chr01	*WmGSB1.1–6*	35,976,680	Intragenic region (Exon)	G	A/A	G/G
	Chr01	*WmGSB1.1–7*	28,931,313	Intragenic region (Intron)	G	T/T	G/G
	Chr01	*WmGSB1.1–8*	34,327,226	Intergenic region	T	C/C	T/T
	Chr01	*WmGSB1.1–9*	35,767,079	Intergenic region	A	C/C	A/A
***ClGSB10.1***	Chr10	*WmGSB10.1–1*	14,128,011	Intergenic region	C	C/C	T/T
	Chr10	*WmGSB10.1–2*	33,750,422	Intragenic region (Exon)	G	T/T	G/G
	Chr10	*WmGSB10.1–3*	33,653,875	Intergenic region	T	C/C	T/T
***ClGSB11.1***	Chr11	*WmGSB11.1–1*	18,454,241	Intergenic region	C	C/C	T/T
	Chr11	*WmGSB11.1–2*	19,699,196	Intergenic region	C	T/T	C/C
	Chr11	*WmGSB11.1–3*	25,592,538	Intergenic region	T	A/A	T/T
	Chr11	*WmGSB11.1–4*	26,099,882	Intergenic region	C	CTCCTTTT/ CTCCTTTT	C/C

### Validation of marker performance

**Fig. 4:**
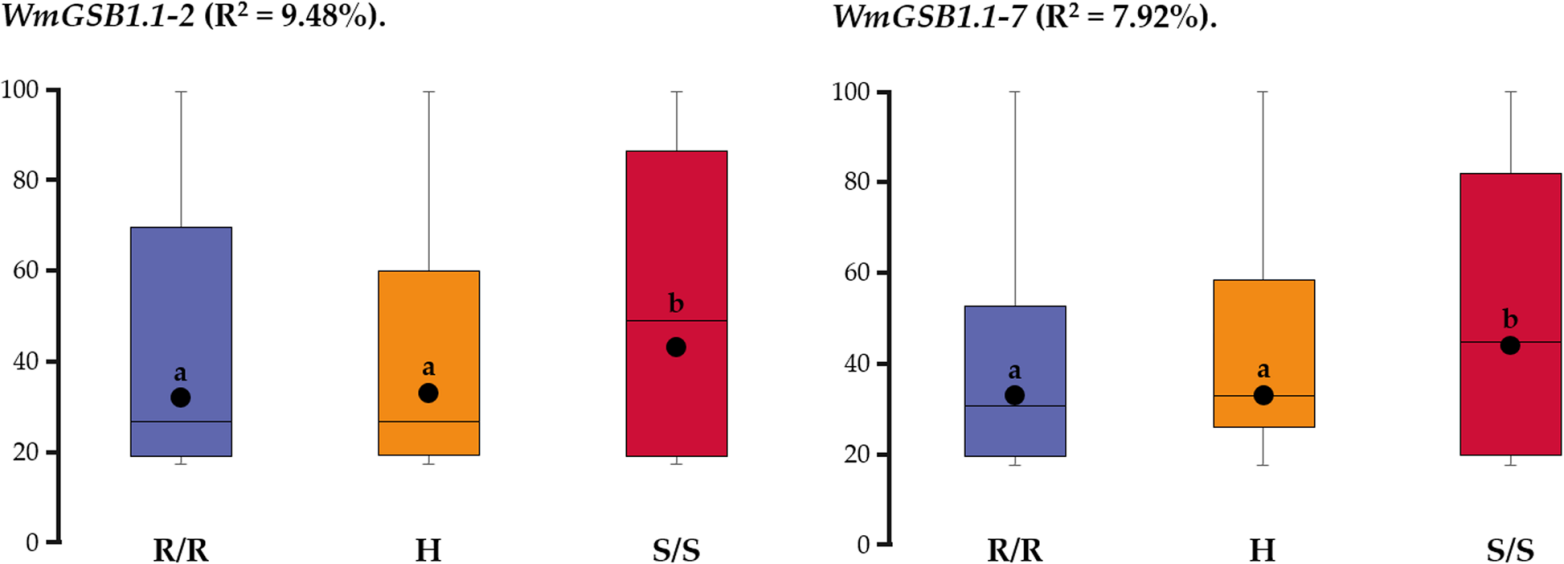
Box plots showing the efficacy of the HRM markers *ClGSB1.1–2* and *ClGSB1.1–7* in distinguishing the resistant and susceptible genotypes in parents, F_1_ and F_2_ population. The whiskers represent the lowest or highest data point within the 1.5 interquartile range of the lower or upper quartile. The thick horizontal lines in the boxes represent the median (*P* < 0.05; one-way ANOVA). R/R genotype represents the resistant (PI 279461) allele, H genotype represents the heterozygote and S/S genotype represents the susceptible (PI 223764) allele. Dots within the box plots indicate means of lines harboring respective alleles

The nine markers, seven within the QTL, *ClGSB1.1* and two within *ClGSB10.1* that clearly distinguished the resistant and susceptible parents and their heterozygous hybrids were further validated using the eight watermelon accessions and F_2_ populations (Table 3). Among these, two markers, WmGSB1.1–2 (Chr01_28,572,939) and WmGSB1.1–7 (Chr01_28,931,313), were found to be highly associated with GSB resistance in watermelon accessions (both match 6 out of 8 accessions), with a highly significant correlation between marker genotyping and F2 population PDI scores (R^2^ value of 9.48 and 7.92%, respectively at *P* < 0.05) (Figs. 4 and S2). However, the genotyping of markers in *ClGSB10.1* didn’t show a strong correlation with PDI scores for resistance to GSB. For *WmGSB1.1–2* and *WmGSB1.1–7* assays, PDI scores were significantly lower for individuals homozygous for the resistant allele (R/R) than that of individuals homozygous for the susceptible allele (S/S) (Fig. 4), whereas other markers didn’t show such significant correlation (data not shown). Genotyping by marker *WmGSB1.1–2* (Chr01_28,572,939) in F_2_ population showed a significant association with PDI scores (RR = 32.8, SS = 44.5, *P* < 0.05) and had an R^2^ value of 9.48%. Marker *WmGSB1.1–7* (Chr01_28,931,313) showed a significant difference (*P* < 0.05) in PDI between RR allele (PDI = 33.64) and SS allele (PDI = 42.8) in F_2_ population (R^2^ = 7.92%).

**Table 3:**
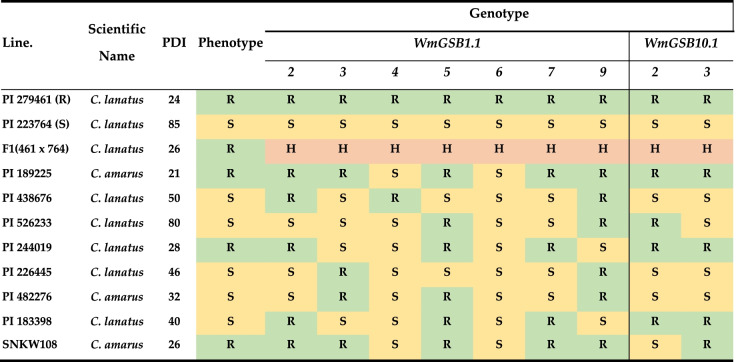
Genotyping efficiency of the selected nine HRM markers in distinguishing GSB resistant vs susceptible individual from diverse genotypes

### Candidate genes identification

**Fig. 5:**
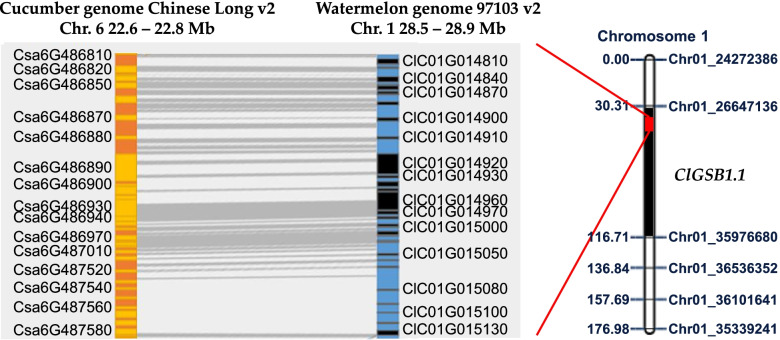
Syntenic analysis of cucumber gummy stem blight-linked quantitative trait locus (QTL) areas in chromosome 6 with the genome of watermelon chromosome 1. Orange and blue represent *Cucumis sativus* (Chinese Long v2) and *Citrullus lanatus* (97,103 v2) genome, respectively

The flanking markers, *WmGSB1.1–2* and *WmGSB1.1–7* of the QTL *ClGSB1.1*, had the highest phenotypic variance and had a significant difference in PDI between the resistant allele (R/R) and the susceptible allele (S/S) (Fig. 4). The distance between these two flanking markers on chromosome 1 is approximately 0.36 Mbp, a region with 40 genes in CuGenDB (http://cucurbitgenomics.org/; accessed on 20 Dec 2021) (Table S6). Among these, the key genes include the necrotrophic fungal disease-related genes, two receptor-like kinase (RLKs) domain-containing genes- *ClC01G014900* and *ClC01G015010*, two WRKY transcription factor genes- *ClC01G014910* and *ClC01G014990* [37–39] and one pathogenic type III effector avirulence factor Avr cleavage site-containing protein- *ClC01G015130* [40–42]. Syntenic analysis revealed conserved synteny between two flanking markers in *ClGSB1.1* and a locus in *Cucumis sativus* (cucumber) chromosome 6 associated with GSB resistance (Fig. 5) [16].

Previously, we reported an expression analysis of forty-four NBS-encoding *R* genes related to GSB resistance [12]. To validation the expression pattern of these NBS genes in PI 279461 and PI 223764, qRT-PCR was used to confirm. An expression analysis of forty-four NBS genes revealed up-regulation of forty-two genes and down-regulation of two genes, at least at one time point after pathogen inoculation in the resistant line compared to that of the susceptible line (Fig. S3). Among the up-regulated genes, *ClC01G015700* was identified to be within the QTL *ClGSB1.1*. On the other hand, five genes, *ClC10G198730*, *ClC10G198620*, *ClC10G199670*, *ClC10G199720,* and *ClC10G198140* were located within *ClGSB10.1* and two genes, *ClC11G216720* and *ClC11G216940* were within *ClGSB11.1* QTL regions (Table S3). *ClC01G015700*, encoded NBS-LRR and RPW8 domain and located 0.52 Mbp downstream of the developed marker *WmGSB1.1–7* within the QTL *ClGSB1.1* (29,450,021 – 29,452,887), was also highly expressed in resistant line and potential candidate gene in our previous study [12]. The disease resistance related domain containing genes within the identified QTL regions including the genes that showed higher expression upon pathogen inoculation may be potential candidate genes for GSB resistance. However, additional research will be required to fine map the region to identify the causal gene(s) upon functional varification.

## Discussion

Identifying the genetic determinants of resistance to GSB is important for developing suitable varieties against the devastating disease. Watermelon accessions ‘PI 189225’ and ‘PI 271778’ [7, 43] and very recently, the accessions ‘PI 279461,’ ‘PI 526233,’ and ‘PI 482283’ [9] have been reported to carry sources of GSB resistance. Resistance to GSB in cucurbits has been identified to be monogenic dominant, monogenic recessive and polygenic previously [14, 15]. For example, GSB resistance in ‘PI 189225’ was regulated by a single gene, *db* [11]. However, new study revealed the involvement of numerous genes with environmental variables for GSB resistance [4]. We identified the inheritance of this trait to be controlled by polygenes as evident from the continuous distribution of the resistance scores and the segregation ratio of resistance and susceptible F_2_ genotypes upon infection with the inoculum of the causal pathogen (Fig. 2B and Table S4). This is in agreement with the findings of several recent studies as well [4, 12–14, 16, 17, 44].

Previous mapping studies have identified several QTLs that regulated resistance to major diseases such as bacterial fruit blotch (BFB), fusarium wilt, and papaya ringspot in watermelon [45–49]. QTLs associated with GSB resistance have been identified in the seedlings [16, 17], and stems [44] of cucumber. Syntenic regions of the known GSB QTL of cucumber were explored on chromosome 9 of melon to explore the potential genes associated with GSB resistance in melon [26]. In watermelon, several QTLs for resistance to GSB have been reported very recently [13–15]. Using a single-nucleotide polymorphism [SNP]-index identified by the bulk sergeant analysis of 211 inter-specific (cultivated *C. lanatus* breeding line K3 ✕ wild *C. amarus* accession PI 189225) F_2:3_ plants upon inoculation with a single isolate of *S. cucurbitacearum*, a major QTL, *Qgsb8.1* spanning a region of 5.7 Mb on chromosome 8 was identified for GSB resistance in watermelon genotype, PI 189225 [13]. Gimode et al., 2021 identified three QTLs, *ClGSB3.1*, *ClGSB5.1* and *ClGSB7.1*, based on the SNPs detected by GBS analysis of an F_2:3_ interspecific population derived from Crimson Sweet (*C. lanatus*) ✕ PI 482276 (*C. amarus*) against *Stagonosporopsis citrulli* 12178A in the greenhouse grown seedlings of watermelon. Lee et al., 2021 constructed a linkage map using 113 polymorphic SNPs using Fluidigm® SNP Type™ assays and identified three QTLs, *qSB6.1, qLL8.1,* and *qSB8.1* from an F_2_ population of 178 genotypes from a cross of susceptible line ‘920533’ (*C. lanatus*) ✕ resistant line ‘PI 189225’ (*C. amarus*). These studies identified QTLs for GSB resistance on chromosomes 3, 5, 6, 7, and 8, which have a common donor species, *C. amarus* [14, 15]. Contrastingly, we identified three QTLs, *ClGSB1.1*, *ClGSB10.1*, and *ClGSB11.1*, located on chromosomes 1, 10, and 11, respectively, using parents both belonging to *C. lanatus*. Besides the differences in the location of the QTLs, the phenotypic variation explained by the identified QTLs is also variable, as our QTLs explained only around 10% of phenotypic differences, whereas the previously identified QTLs, *Qgsb8.1* and ClGSB7.1, explained > 30 and > 20% of phenotypic variation, respectively [14, 15]. The phenotypic variation explained by the identified QTLs, *qLL8.1*, and *qSB8.1*, however, explained similar percentages (9.7–10.5%) of phenotypic variations. These differences in the location of QTLs and the percentages of phenotypic variations explained may be due to the use of different species as sources of resistance, geographically different and differentially aggressive pathogenic isolates, different methods of inoculation and disease assessment, and differential marker density in QTL mapping.

In this study, one major QTL, *ClGSB1.1*, was found on watermelon chromosome 1 at 8.89 Mbp within the flanking markers, *WmGSB1.1–2* and *WmGSB1.1–7*, spanning 0.36 Mbp region (Table 2). Within this QTL, a total of 40 genes were identified in *ClGSB1.1* region, of which five genes including two RLKs (*ClC01G014900* and *ClC01G015010*), two WRKY transcription factors (*ClC01G014910* and *ClC01G014990*), and one AvrRpt-cleavage domain protein (*ClC01G015130*) are predicted to be associated with GSB resistance. Many RLK protein have been shown to belong to a candidate resistance gene for necrotrophic pathogen [12, 50]. Besides, WRKY transcription factor 75 was found to positively modulate jasmonate-mediated plant defenses against necrotic fungal pathogens [38, 39, 51]. The C-terminal domains of WRKY, AvrRpt-cleavage site, and protein kinase genes belong to a family of proteins that play critical roles in plant defense signaling [52–54]. The majority of R genes in plants encode proteins with a nucleotide-binding site (NBS) and leucine-rich repeats (LRRs), both of which are essential for plant–pathogen recognition. Forty-four NBS-encoding *R* genes were previously identified in watermelon based on genome version 1 of accession 97,103 [12, 19]. We also identified the equivalent number of NBS genes in 97,103 genome version 2 [31] and analyzed their expressions upon pathogen inoculation. Up-regulation of the genes within the QTL regions such as *ClC10G198730*, *ClC10G198620*, *ClC10G199670*, *ClC10G199720,* and *ClC10G198140* within *ClGSB10.1; ClC11G216720* and *ClC11G216940* within *ClGSB11.1;* and especially, the highly expressed gene *ClC01G015700* within the QTL *ClGSB1.1 (*located only 0.52 Mbp downstream of the developed marker *WmGSB1.1–7)* are putative candidates. All these genes thus could be the key genes playing important roles in conferring resistance to GSB.

Developing high-throughput molecular markers that can be used for mass screening is essential for success in modern breeding programs as it can reduce the labor-intensive and time-consuming phases of the breeding process and can offer precise genotyping based selection. In this study, we developed and validated two HRM markers, *WmGSB1.1–2* and *WmGSB1.1–7*, linked to QTL *ClGSB1.1* on chromosome 1, which showed high correlation with the phenotypic difference in PDI in F_2_ population. Besides, other markers, *WmGSB1.1–*3, *WmGSB1.1–*4, *WmGSB1.1–5, WmGSB1.1–*6, and *WmGSB1.1–*9, linked to the same QTL also showed reasonable correlation with the phenotypic variation in F_2_. These HRM markers are advantageous from other recently developed KASP markers as the HRM markers are robust to PCR inhibition and thereby, results in fewer miscalled alleles and increased accuracy of results. Besides, those KASP markers were designed based on the QTLS found in different populations with resistance derived mainly from *C. amarus* against isolates of various origins [13, 14]. Whereas, the source of resistance in our population was derived from *C. lanatus*. Yet, our HRM markers were found to be effective in screening resistance alleles of *C. amarus* as well (Fig. 3). So, the developed HRM markers can be used in developing varieties with high resistance to GSB. Future studies should be focused on fine mapping and functional analyses of key R-genes that may offer durable resistance against diverse pathogenic strains, which can be used to further improve the resistance to GSB in watermelon.

## Conclusion

In this study, we identified three QTLs, *ClGSB1.1*, *ClGSB10.1*, and *ClGSB11.1* on chromosomes 1, 10 and 11, respectively, using an F2 population derived from two *C. lanatus* cultivars, ‘PI 279461’ and ‘PI 223764’, and several key candidate R-genes. Two HRM markers linked to GSB resistance in *ClGSB1.1* were also developed and validated using diverse watermelon lines and F_2_ population. This will be helpful in breeding programs aimed at improving GSB resistance in watermelon.

## Supplementary Information

**Additional file 1.**

**Additional file 2.**

## Data Availability

The raw sequence data from this study have been deposited in the publicly accessible National Center for Biotechnology Information (NCBI, https://www.ncbi.nlm.nih.gov/) database as PRJNA804585. The datasets supporting the conclusions of this article are included within the article and its additional files.
