# Supplementary material for: QTL associated with Gummy Stem Blight (GSB) resistance in watermelon
Source: BMC Genomics. 2022 Sep 3;23:632. doi: 10.1186/s12864-022-08849-2 (PMC9441027; doi:10.1186/s12864-022-08849-2)
Supplement: Supplementary file 1 — Additional file 1. [file 12864_2022_8849_MOESM1_ESM.pdf]

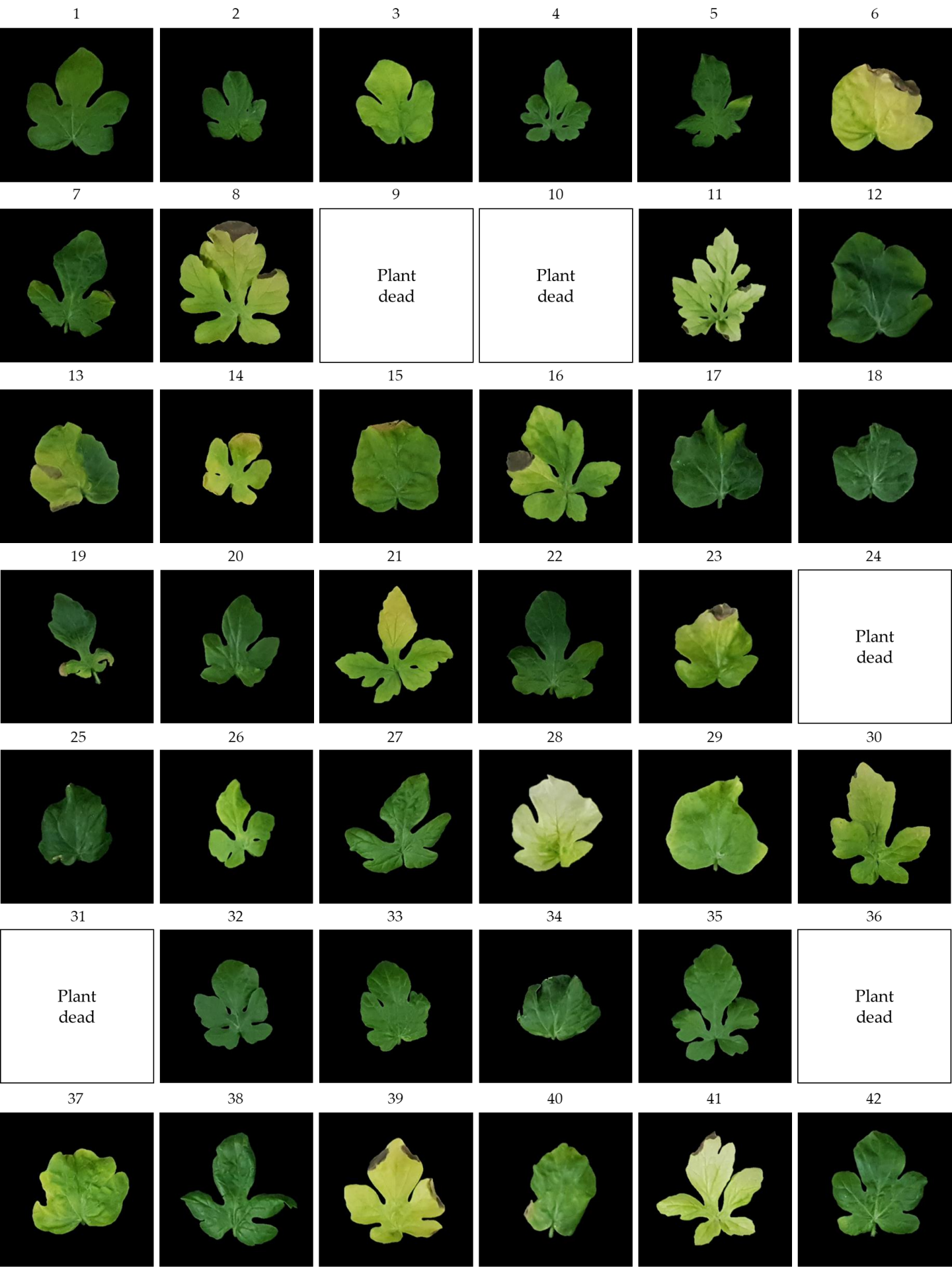

Figure S1. Disease symptoms of watermelon F2 populations two weeks after being inoculation with *Didymella bryoniae*

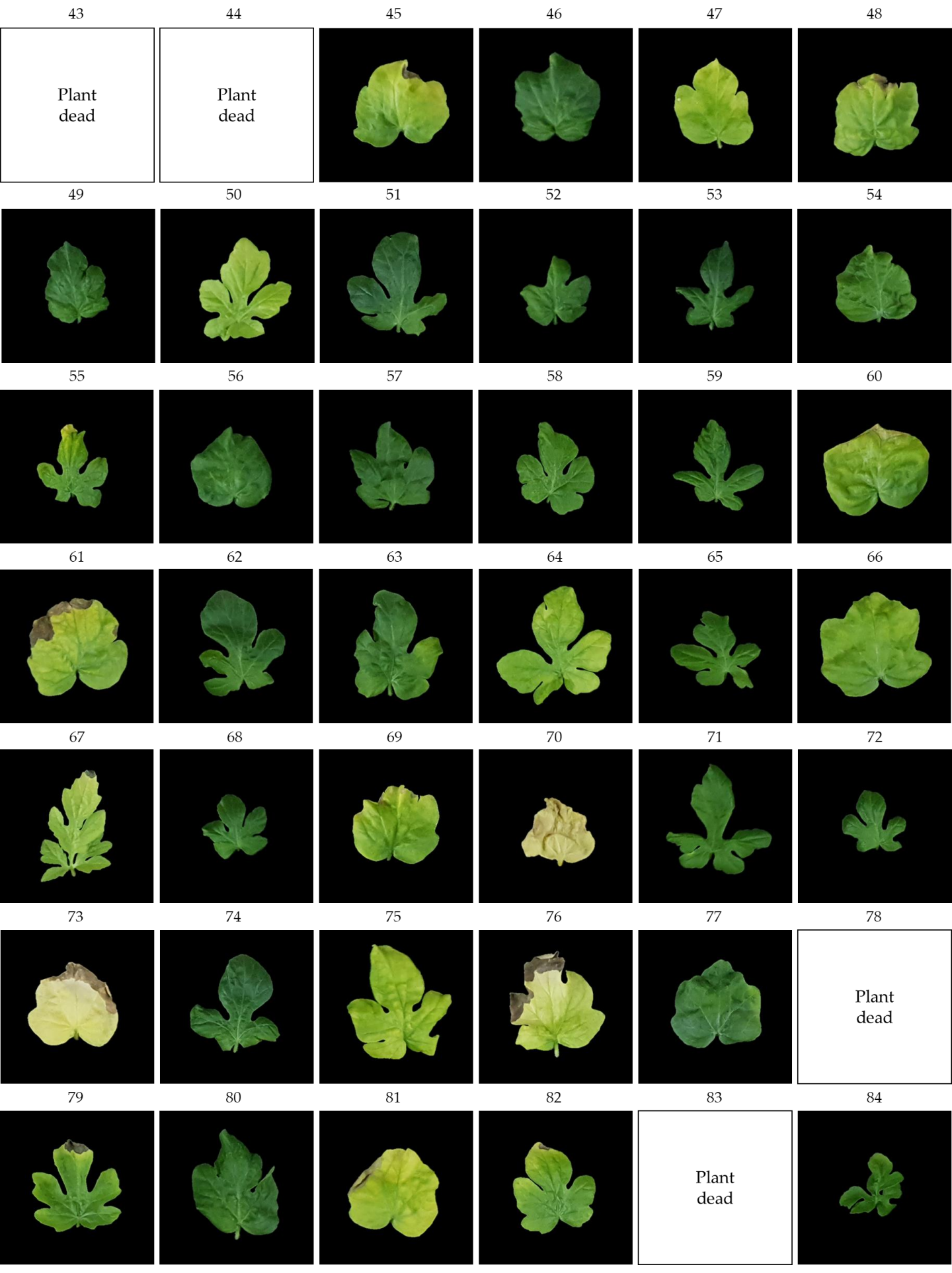

Figure S1. Continued

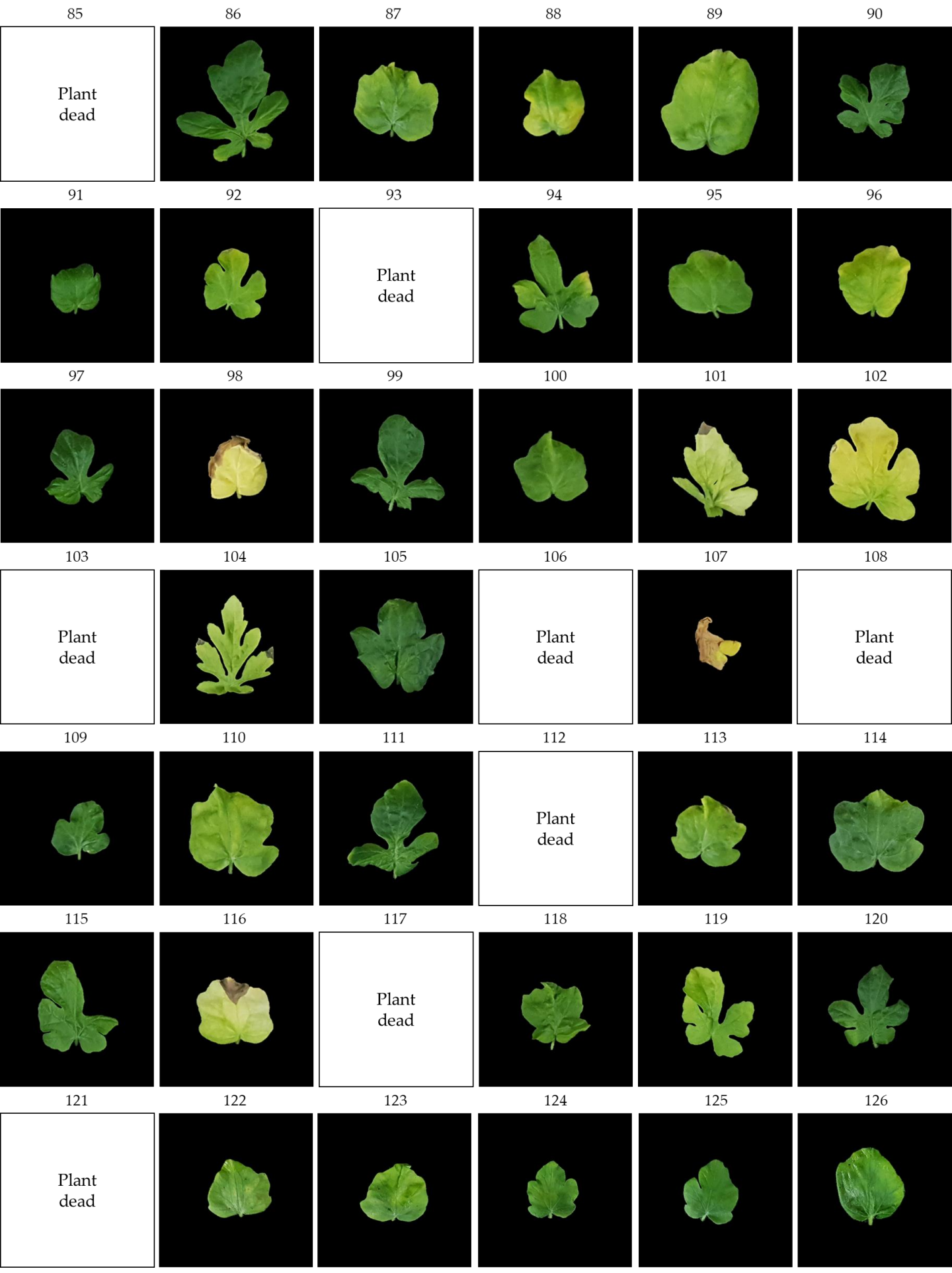

Figure S1. Continued

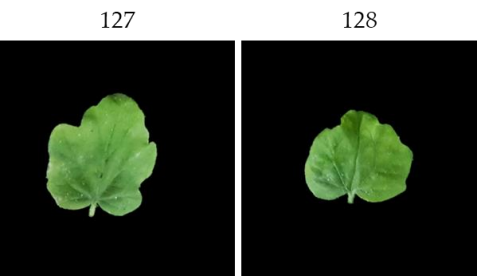

Figure S1. Continued

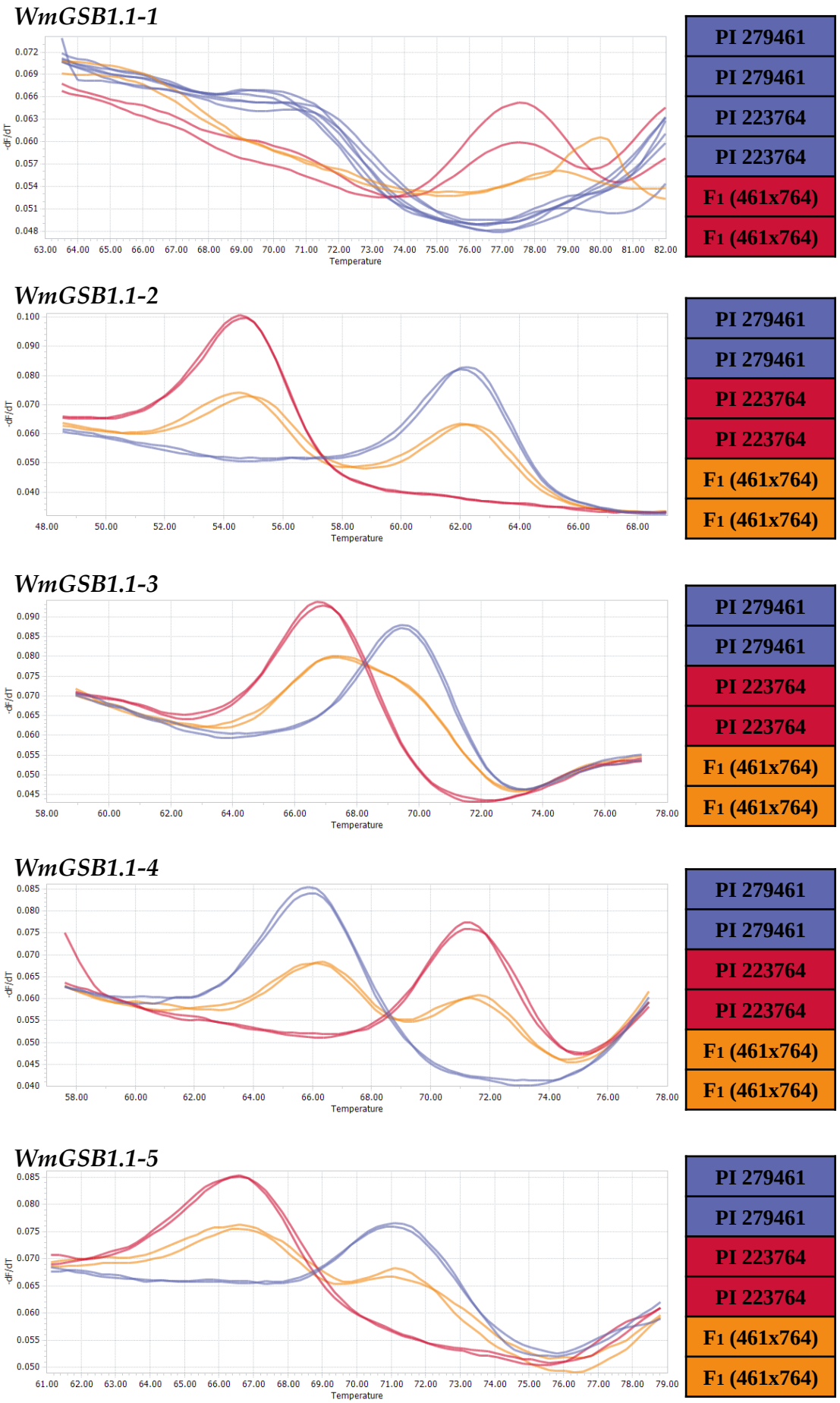

Figure S2. Melting curves of HRM markers in *WmGSB1.1*, *WmGSB10.1* and *WmGSB11.1* developed in this study. Melting curves present the form of normalized melting peaks curve. Blue curves, Plants of 'PI 279461' genotype; Red curves, Plants of 'PI 223764' genotype;Orange curves, heterozygous plants.

WmGSB1.1-6

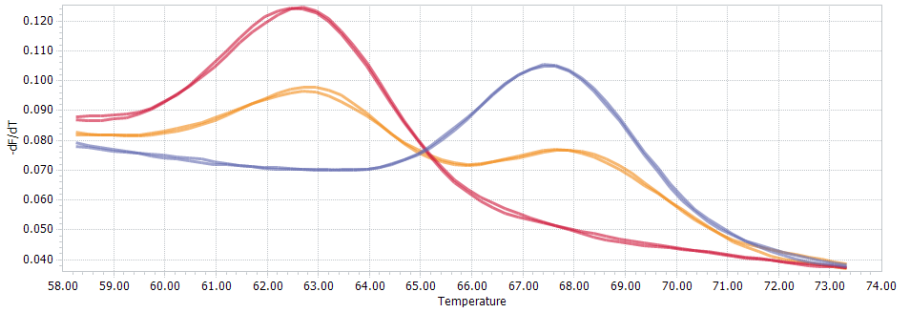

PI 279461
PI 279461
PI 223764
PI 223764
F1 (461x764)
F1 (461x764)

WmGSB1.1-7

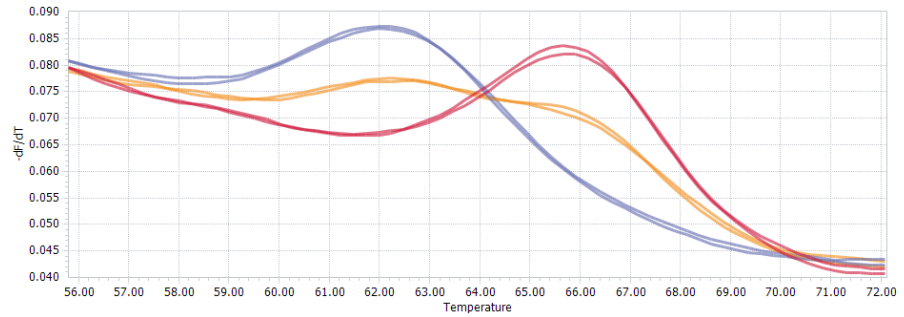

PI 279461
PI 279461
PI 223764
PI 223764
F1 (461x764)
F1 (461x764)

WmGSB1.1-8

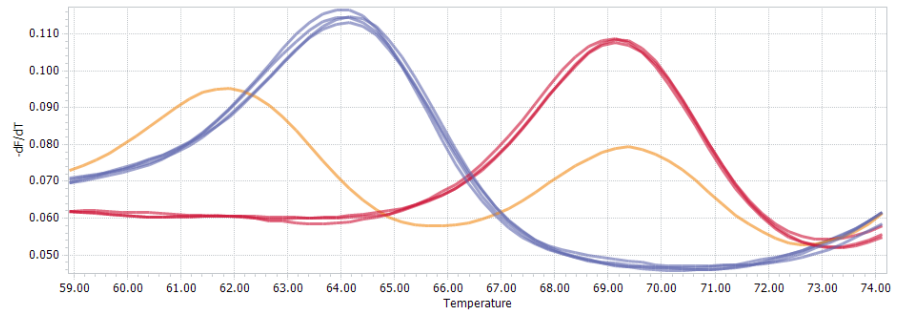

PI 279461 #1
PI 279461 #2
PI 223764 #1
PI 223764 #2
F1 (461x764)
F1 (461x764)

WmGSB1.1-9

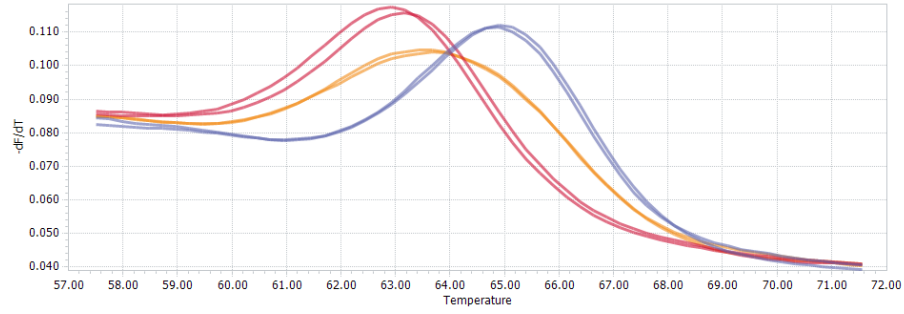

PI 279461
PI 279461
PI 223764
PI 223764
F1 (461x764)
F1 (461x764)

WmGSB10.1-1

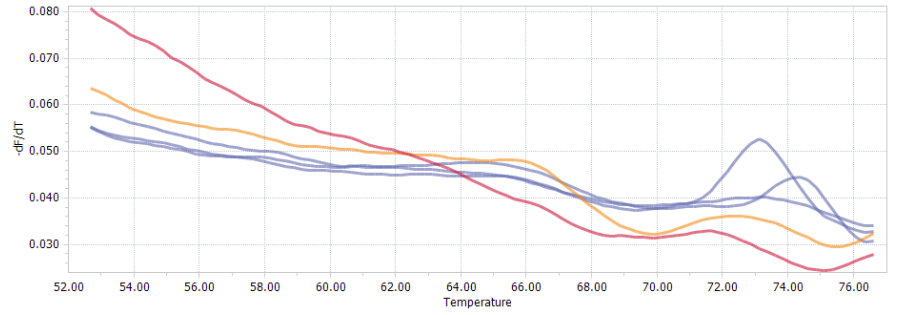

PI 279461 #1
PI 279461 #2
PI 223764 #1
PI 223764 #2
F1 (461x764)
F1 (461x764)

Figure S2. Continued.

WmGSB10.1-2

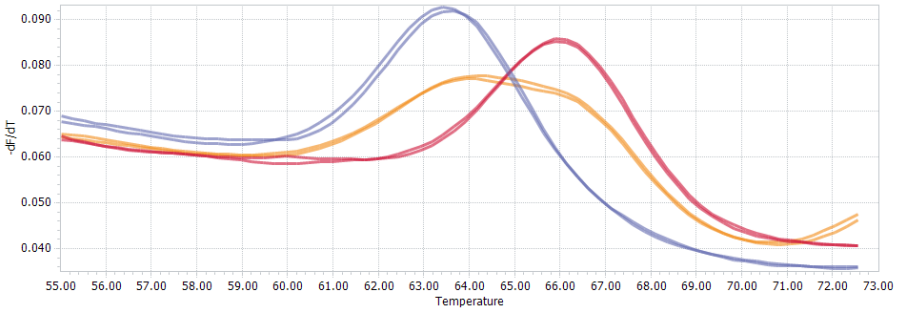

PI 279461
PI 279461
PI 223764
PI 223764
F1 (461x764)
F1 (461x764)

WmGSB10.1-3

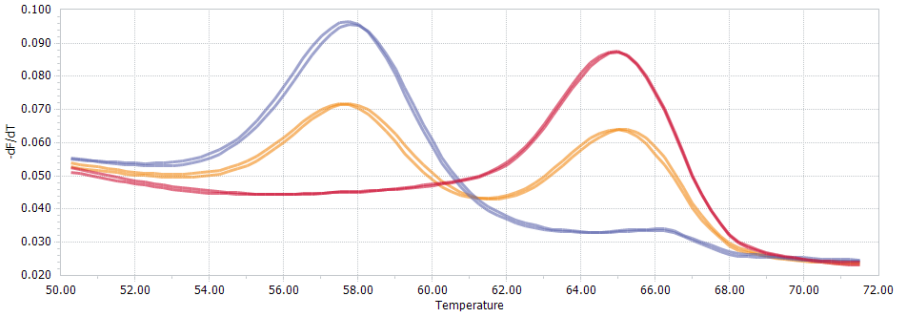

PI 279461
PI 279461
PI 223764
PI 223764
F1 (461x764)
F1 (461x764)

WmGSB11.1-1

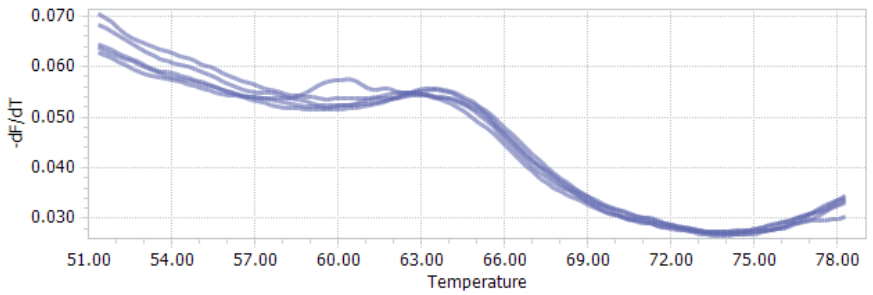

PI 279461 #1
PI 279461 #2
PI 223764 #1
PI 223764 #2
F1 (461x764)
F1 (461x764)

WmGSB11.1-2

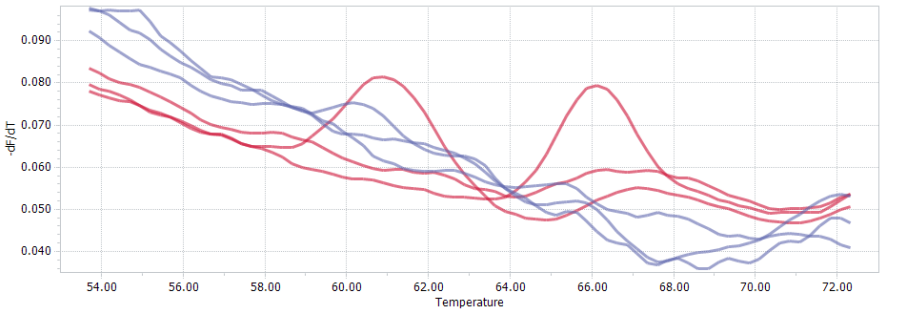

PI 279461
PI 279461
PI 223764
PI 223764
F1 (461x764)
F1 (461x764)

WmGSB11.1-3

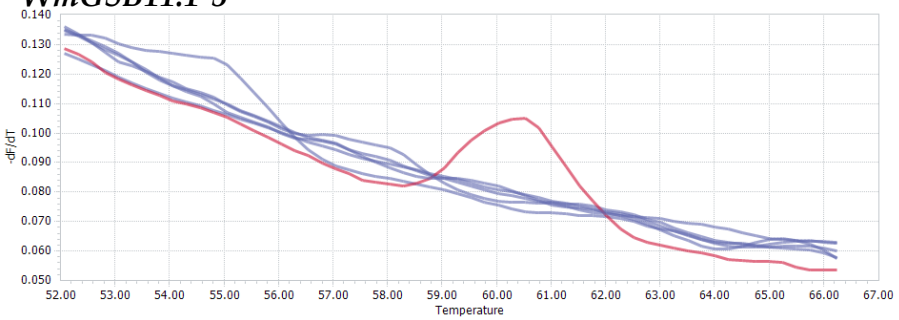

PI 279461 #1
PI 279461 #2
PI 223764 #1
PI 223764 #2
F1 (461x764)
F1 (461x764)

Figure S2. Continued.

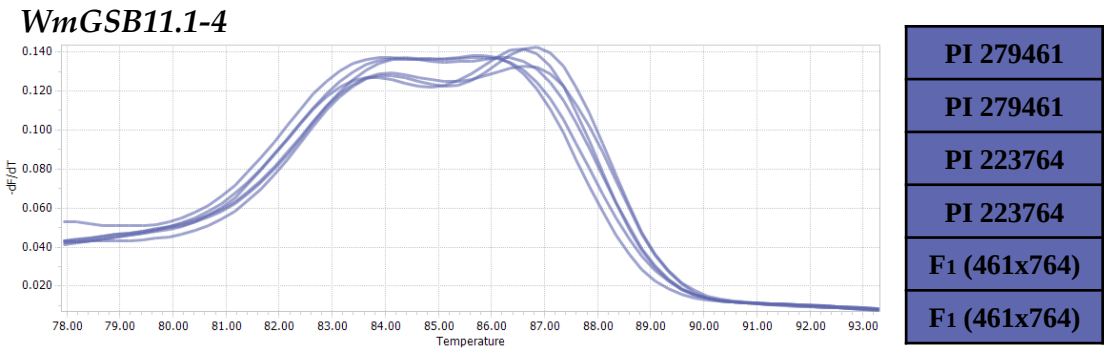

Figure S2. Continued.

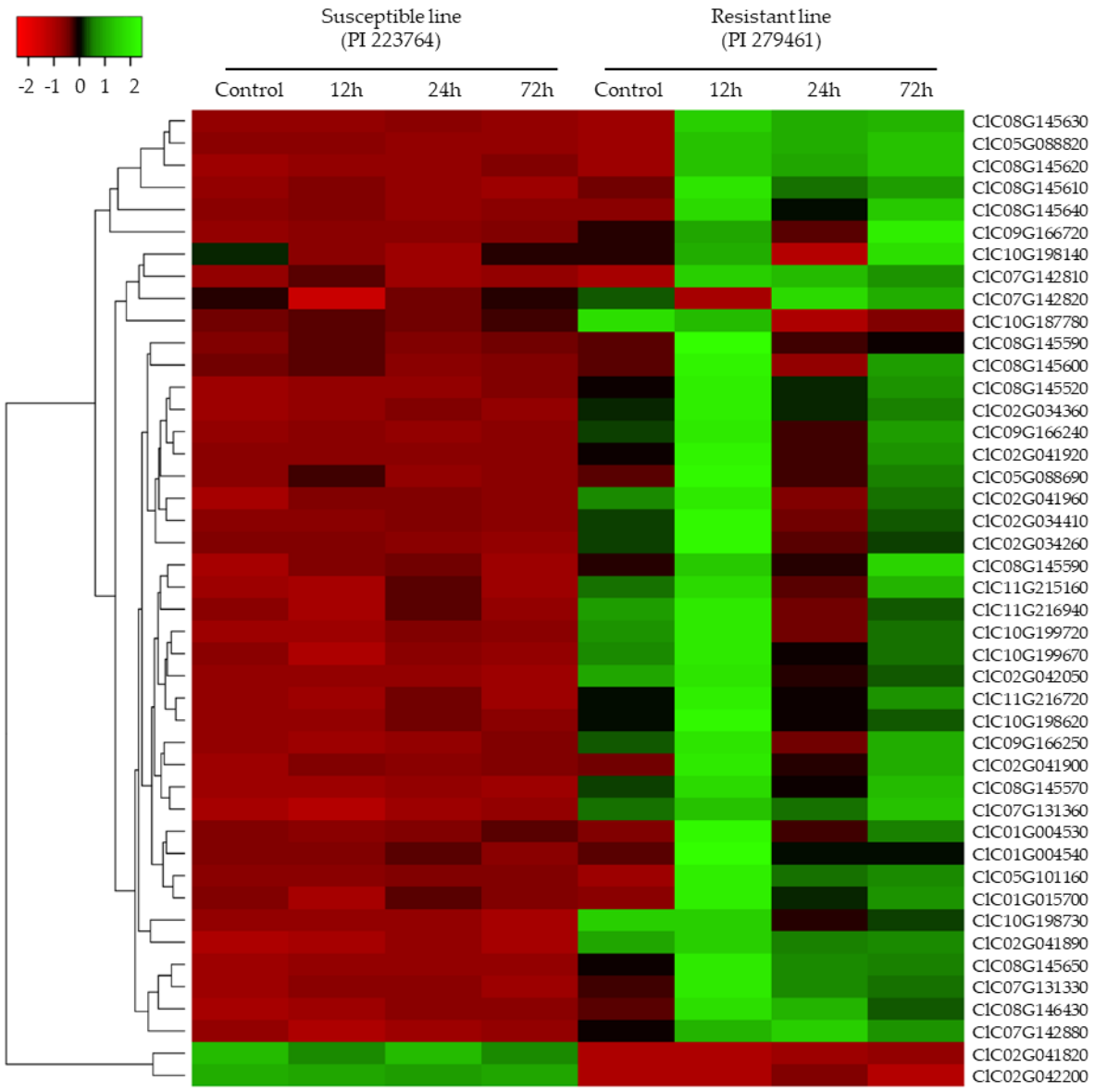

Figure S3. The expression patterns of the 44 NBS-encoding R genes determined by qRT-PCR in gummy stem blight resistant and susceptible watermelon lines infected with *D. bryoniae* at various times are depicted in a heat map. Green. Upregulation; red, downregulation; black, intermediate.
